# T Regulatory Cells and Priming the Suppressive Tumor Microenvironment

**DOI:** 10.3389/fimmu.2019.02453

**Published:** 2019-10-15

**Authors:** Christina M. Paluskievicz, Xuefang Cao, Reza Abdi, Pan Zheng, Yang Liu, Jonathan S. Bromberg

**Affiliations:** ^1^Department of Surgery, University of Maryland School of Medicine, Baltimore, MD, United States; ^2^Department of Microbiology and Immunology, University of Maryland School of Medicine, Baltimore, MD, United States; ^3^Division of Renal Medicine, Transplantation Research Center, Brigham and Women's Hospital, Harvard Medical School, Boston, MA, United States; ^4^Institute of Human Virology, University of Maryland School of Medicine, Baltimore, MD, United States; ^5^Center for Vascular and Inflammatory Diseases, University of Maryland School of Medicine, Baltimore, MD, United States

**Keywords:** Treg, immunosuppression, tumor microenvironment, metastasis, anti-tumor immunity

## Abstract

Treg play a central role in maintenance of self tolerance and homeostasis through suppression of self-reactive T cell populations. In addition to that role, Treg also survey cancers and suppress anti-tumor immune responses. Thus, understanding the unique attributes of Treg-tumor interactions may permit control of this pathologic suppression without interfering with homeostatic self-tolerance. This review will define the unique role of Treg in cancer growth, and the ways by which Treg inhibit a robust anti-tumor immune response. There will be specific focus placed on Treg homing to the tumor microenvironment (TME), TME formation of induced Treg (iTreg), mechanisms of suppression that underpin cancer immune escape, and trophic nonimmunologic effects of Treg on tumor cells.

## Introduction

Natural Treg (nTreg), induced by self-antigens in the thymus, home to sites of tumors, while iTreg, induced by antigens in the periphery, are created as a result of a specific cytokine milieu of the TME. Tregs are a subset of CD4+ T cells that are distinguished from immune cells through expression of Forkhead box protein 3 (FoxP3) transcription factor. FoxP3 stabilizes the suppressive phenotype and capabilities of Treg. FoxP3 mutations or knockout result in fatal lymphoproliferative conditions, and autoimmune pathology secondary to uncontrolled activation of CD4+ T cells (1–3). The subsets of nTreg, and iTreg are difficult to distinguish *in vivo*, although there are several markers that are useful in determining the origin of the cell type (4). Helios is a member of the Ikaros family, and is a transcription factor critical in lymphocyte development and homeostasis (5). Neuropilin-1 (Nrp-1), a semaphorin III receptor, serves as a transmembrane glycoprotein for isoforms of vascular endothelial growth factors (VEGFs), endothelial growth factors, and transforming growth factor beta (TGFβ) (6). Helios and Nrp1 can be expressed on cell surfaces as a consistent marker of nTreg origin and activation (5, 7, 8). Although there is limited evidence demonstrating differences in antigen recognition between nTreg and iTreg, it is plausible to consider nTreg specificity in recognition of self-antigen expressed on tumor cell surfaces, whereas iTreg may specifically recognize *de novo* antigens (9). CD4+CD25+ FoxP3+ nTreg and iTreg express characteristic receptors including cytotoxic T-lymphocyte-associated protein 4 (CTLA-4), glucocorticoid-induced TNFR-related gene (GITR), and CD25 (IL-2 receptor α-chain) which further differentiate from other immune cells, and which mediate immunosuppressive functions (10, 11).

In the TME, Treg of either origin employ unique mechanisms to mediate immunosuppression and cancer progression. There is cross-talk among Treg and the other cells in the TME, including infiltrating lymphocytes, stromal cells, and tumor cells (12). Treg employ several immunologic mechanisms including inhibition of antigen presenting cell (APC) maturation, secretion of inhibitory cytokines, and production of cytotoxic granzyme and perforin (4). Aside from immunologic mechanisms deployed by Treg responding to cancer, potential nonimmunologic support is provided to tumors through novel interactions including potentiation of angiogenesis (13, 14), tumor growth (15), and proliferation, and tumor transition to metastatic disease (16, 17).

Therefore, Treg recruitment, induction, and maintenance in the TME play protean roles in inhibition of anti-tumor responses and progression of malignancy. An understanding of the relationship between Treg and tumor cells will derive benefits for patient and disease specific treatments.

## Recruitment of Natural Treg to the TME

nTreg homing is a critical step in initiation and propagation of the immunosuppressive TME (18). There are numerous examples of cytokine gradients established both by tumor and immune cells that serve as driving forces of nTreg entry into the TME.

**Figure 1:**
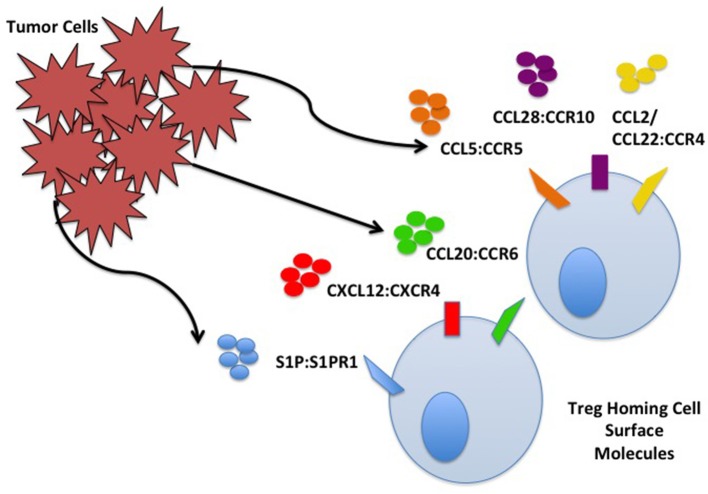
Tumor cell and Treg homing interactions. Treg home to the TME through interactions with chemokines/ligands produced by TME components including cancer cells. Some interactions are depicted including S1P:S1PR, CXCL12:CXCR4, CCL20:CCR6, CCL5:CCR5, CCL28:CCR10, and CCL2/22:CCR4.

Tan demonstrated that nTreg in the TME of Pan02 pancreatic tumors increase in comparison to the percentage of nTreg in spleen and non-tumor draining lymph nodes (LNs). nTreg have increased CCR5 expression, and Pan02 tumors produce a 4-fold increase in CCL5 compared to pancreatic tissue controls. CCL5 knockdown results in significant decrease in infiltrating nTreg compared to wild type Pan02. Systemic CCR5 antagonist administration results in delayed tumor growth, increased survival, and decreased infiltrating nTreg in the TME (18). Similar CCR5/CCL5 dependent recruitment of nTreg to the TME has been demonstrated in other cancer models including breast, colorectal, prostate, and lung (19–21). Myeloid derived suppressor cells (MDSCs) are found in tumor tissue of RMA-S lymphoma, including monocytic myeloid derived suppressor cells (MO-MDSCs). The MO-MDSCs secrete CCL5 (22). Treg migrate toward tumor tissue MO-MDSC, and migration is inhibited in CCR5 knockout Treg, leading to decreased tumor nTreg, delay in tumor growth, and improved outcomes (22). nTreg homing interactions in the TME are explained in Figure 1.

The TME can be toxic to some effector lymphocytes secondary to hypoxia from rapidly dividing tumor cells outgrowing their blood and nutrient supply; Tregs migrate toward this environment where they further carry out suppressive functions. Tumor cells use hypoxic conditions to promote homing of nTreg. Facciabene et al. demonstrated that human ovarian cancer cells incubated in hypoxic conditions upregulate expression of CCL28, controlled by hypoxia inducible factor 1α (HIF-1α). Supernatants with increased expression of CCL28 result in increased migration of CD4+CD25+FoxP3+ nTreg compared to normoxic supernatants (14). Migrating nTreg express CCR10, the receptor for CCL28. nTreg migration is inhibited with neutralizing antibody to CCL28 or CCR10. Ovarian tumors transduced to overexpress CCL28 (ID8-ccl28) have increased intratumoral and ascitic fluid accumulation of nTreg (14). Intra-peritoneal administration of anti-CCR10 immunotoxin decreases tumor growth through inhibition of Treg migration.

CCR4 and CCL22 facilitate trafficking of nTreg to the TME. CD4+CD25+ nTreg are present within malignant ascites and solid tumor burden of human ovarian carcinomas (23). These nTreg express CCR4 which serves as the receptor for CCL22 and CCL17. Ovarian carcinoma, in addition to gastric, esophageal, breast, lung, and head and neck cancer produce large quantities of CCL22 (24–27). *In vitro* analysis demonstrated a significant decrease in nTreg migration after administration of anti-CCL22 antibody. No change in migration occurs with administration of anti-CCL17 antibody. Similar findings are observed *in vivo* when humanized mice are inoculated with human ovarian tumors with concurrent transfer of human nTreg. Human nTreg migrate to ascitic fluid and solid tumors in a CCL22/CCR4 dependent manner demonstrated by *in vivo* blockade of trafficking after administration of monoclonal antibody (mAb) to CCL22 (23). Mice inoculated with ovarian cancer cell lines expressing CCL22 accumulate CCR4+ nTreg, which are blocked by administration of anti-CCR4 mAb, further inhibiting tumor size and progression (28).

Numerous other interactions have been characterized between nTreg and TME chemokines that facilitate nTreg homing and immunosuppressive functions (Table 1). Tumor derived sphingosine-1-phosphate (S1P) is tumor protective through nTreg S1P receptor-1 (S1PR1) (29). E0771 breast cancer and B16 melanoma show TME accumulation of nTreg via S1P/S1PR1 (30). Genetic and pharmacologic blockade of S1PR inhibits nTreg accumulation in tumors and slows growth (30). Human malignant glioma lines and fresh tumor tissue express elevated CCL2, and nTreg obtained from patient samples express elevated CCR4. Treatment of tumors with chemotherapeutic agents temozolomide or bis-chloroethylnitrosourea inhibits CCL2 production and FoxP3+ Treg migration *in vitro* (31). CXCL12 produced by tumor cells attract CXCR4+ Treg in addition to MDSC and plasmacytoid dendritic cells (pDCs) (32). Treatment of mice inoculated with the ovarian cancer line BR5-1, with a specific antagonist for CXCR4, leads to increased tumor death, tissue necrosis, decreased intra-peritoneal disease burden, increased recruitment of tumor specific effector T cells, reduction in intratumoral Treg, and significantly increased survival (33). In breast cancer patients, the TME is enriched with CCR6+ Treg; increased enrichment of these Treg is more commonly associated with later disease stage and poorer prognosis (34). In hepatocellular carcinoma (HCC), similar findings were demonstrated with increased CCR6+ Treg in the TME, which were inhibited with neutralizing mAb to CCL20. CXCR3+ Treg home to the TME in response to established CCL9/10/11 chemokine gradients. CXCR3+ Treg have been isolated from the TME of ovarian cancer and HCC (37); increased CXCR3+ Treg presence in the TME has been correlated to a blunted effector response, and HCC tumor recurrence following transplantation (38, 39). CCL10^–/–^ and CXCR3^–/–^ mice had decreased recruitment and mobilization of Treg to HCC tumor burden (38).

**Table 1:** nTreg chemotactic ligand and receptor interactions in the TME.

**Chemokine/Ligand**	**Receptor**	**Cancer model**	**References**
S1P	S1PR1	Breast, melanoma	(29, 30)
CCL2	CCR2/CCR4	Glioma	(31)
CXCL12	CXCR4	Ovarian	(32, 33)
CCL20	CCR6	Breast, HCC	(34, 35)
CCL19/CCL21	CCR7	Melanoma	(36)
CCL9/10/11	CXCR3	Ovarian, HCC	(37–39)
CCL22	CCR4	Ovarian, breast, gastric, esophageal	(23–27)

*nTreg express a number of receptors that allow for interaction with chemokines/ligands produced by constituents of the TME*.

Despite a wide body of evidence outlining the extensive mechanisms by which nTreg enter the TME, little evidence is in place regarding which of these mechanisms is most frequently used or more dominant. Therefore, this is a potential avenue for ongoing future research, but presents a gap in the current body of literature. Treg prevalence in the TME is associated with advanced tumor progression and poorer outcomes (35). Taken together, these interactions establish substantial populations of nTreg in the TME that facilitate tumor survival through chemotactic migration and subsequent inhibition of the anti-tumor response.

## Induction of iTreg in the TME

**Figure 2:**
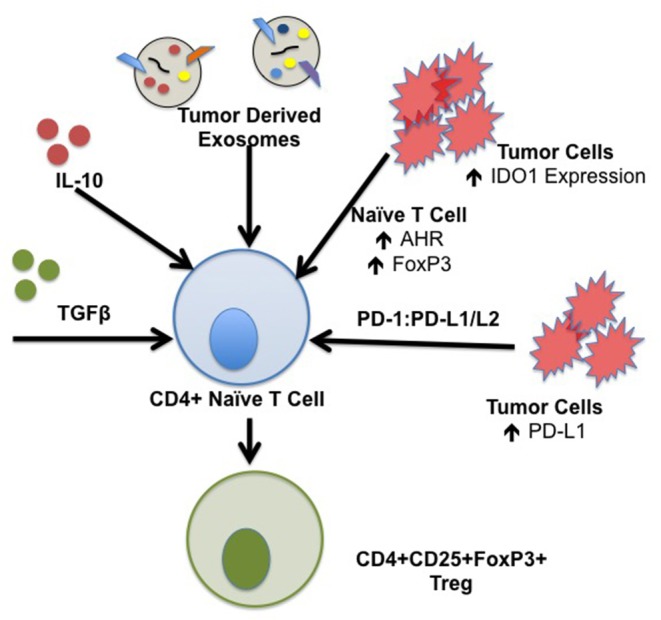
Mechanisms of iTreg induction in the TME. iTreg are derived from naïve CD4+ T cells following exposure to a specific cytokine milieu. Constituents of the TME are capable of producing several factors that form iTreg which further promote immunosuppression and inhibition of the anti-tumor immune response. Specific factors that drive the formation of iTreg include TGFβ and IL-10; TGFβ and IL-10 transcripts have been isolated from tumor subtypes including ovarian, breast, renal cell, lung, and squamous cell carcinoma. Tumor derived exosomes contain IL-10, TGFβ, and Fas ligand capable of generating iTreg. IDO1 is ubiquitously expressed by components of the TME, including tumor cells, stromal cells, DCs, and MDSCs which drive induction of iTreg. Binding of tumor derived PD-L1/L2 to T cell PD-1 is implicated in development, maintenance, and suppressive function of iTreg through stabilization of FoxP3 expression.

iTreg are derived from naïve CD4+ T cells in the periphery in response to tumor stimuli that drive differentiation toward a regulatory phenotype. IL-10 plays a central role in induction of iTreg in the TME. IL-10 is a suppressive cytokine that functions by down-regulating excessive inflammatory responses; IL-10 mRNA transcripts have been isolated from tumor tissues including ovarian, breast, renal, lung, and skin cancer (40). Reports demonstrate constitutive production of IL-10 *in vitro* from several cancer cell lines including colon, lung, and skin carcinoma. IL-10 in the TME is derived from several components including tumor cells in addition to infiltrating leukocytes including T and B cells, macrophages, and NK cells (41). TGFβ also induces iTreg and cancer progression. As tumors grow, they secrete increasing quantities of TGFβ in an autocrine manner (42). The dense stromal network surrounding the tumor and infiltrating immune cells also serve as a source of this cytokine (43, 44). Increased TGFβ in the TME correlates with more advanced stage disease and poorer prognosis (45). Together, TGFβ and IL-10 potentiate the differentiation of human iTreg with increased expression of FoxP3 and CTLA-4 (46). Naïve CD4+CD45RO-CD25- T cells stimulated with anti-CD3/CD28, IL-2, and TGFβ in the presence of IL-10 results in a significant increase in the percentage of FoxP3+ cells and expression of CTLA-4 (47). Induction of iTreg in the TME drives tumor growth, and differentiation of iTreg in the TME is associated with overall poorer patient survival in cancer subtypes. Mechanisms of iTreg induction are summarized in Figure 2.

### Tumor-Derived Extra-Cellular Vesicles

Tumors can produce and secrete vesicular membrane derived material defined as exosomes and microvesicles (MVs) that have potent immunoregulatory properties capable of expanding iTreg and enhancing Treg suppressor function (48, 49). Characterization of MV derived from sera of head and neck squamous cell carcinoma (HNSCC) patients and ascites from ovarian carcinoma patients demonstrates increased expression of IL-10, TGFβ, and Fas ligand (FasL) (49). When MVs isolated from patient sera or ascites are co-incubated with purified CD4+CD25- T cells, the percent of CD4+CD25+FoxP3+ T cells increases in a dose dependent fashion (49). Similarly, tumor derived MVs from both HNSCC and melanoma sera induce *in vitro* expansion of CD4+CD25+FoxP3+ with enhanced suppressor function (50). Huang et al. demonstrated that non-small cell lung cancer (NSCLC) tissue consistently contain MVs with increased levels of epidermal growth factor receptor (EGFR) which induce tolerogenic indoleamine 2,3-dioxygenase 1 (IDO1) producing dendritic cells (DCs). The exact mechanism requires further investigation, but it is believed that DCs are induced through EGFR activation of PI3K leading to upregulated expression of IDO1 in tolerogenic DCs. These IDO1 expressing DCs induce tumor specific iTreg capable of suppressing tumor protein specific CD8+ T cell activity (51). Thus, tumor cells directly and indirectly induce highly suppressive iTreg within the TME via MV.

### Checkpoint Molecules

Interactions between the Programmed Death Receptor (PD-1) and Programmed Death Ligands (PD-L1 and PD-L2) play roles in both development and sustaining iTreg. Interactions between PD-1/PD-L1 or PD-L2 are immune checkpoints that function in peripheral immune tolerance. Signaling by PD-L1 and PD-L2, which are commonly highly expressed on tumor cells and stromal cells in the TME, impairs infiltrating T lymphocyte responses through induction of anergy, exhaustion, and apoptosis upon engagement with the PD-1 receptor. Tumor derived PD-L1 is a potent immunomodulatory mechanism that confers the ability to suppress host T cell immunity (52). PD-1/PD-L1 or PD-L2 interactions are also implicated in development, maintenance, and suppressive function of iTreg through stabilization of FoxP3 expression (53). Francisco demonstrated that PD-L1 synergizes with TGFβ to promote naïve T cell conversion to iTreg; wild type APCs cultured with naïve CD4^+^CD62L^hi^FoxP3 GFP^−^ T cells, anti-CD3, and TGFβ result in a greater percentage of naïve CD4+ conversion to iTreg in comparison to PD-L1^−/−^ APCs. Co-culture of naïve CD4+ cells with PD-L1 Ig coated beads results in increased iTreg conversion (53). Unger et al. demonstrated that treatment of murine bone marrow derived DCs with 1α,25-dihydroxyvitamin D3 (VD3) results in a tolerogenic phenotype including increased PD-L1 expression. Priming CD4+CD25-T cells with VD3-DCs results in potent, suppressive iTreg (54). These data demonstrate a central role for PD-1 and PD-L1/PD-L2 signaling in induction and maintenance of iTreg in the TME.

### IDO

IDO1 is an intracellular rate limiting enzyme that converts tryptophan (Trp) to kynurenine (KYN) (55). IDO1 is silent in tissues in homeostatic conditions; however, it becomes upregulated in pathologic disorders including cancer to facilitate resolution of inflammation (56). Pro-inflammatory cytokines in the TME including IFNγ, TNFα, TGFβ, and IL-6 are potent inducers of IDO1 (56). IDO1 is ubiquitously expressed by components of the TME, including tumor cells, stromal cells, DCs, and MDSCs. Immune suppression via IDO is multifactorial through Trp starvation, direct toxic effects of KYN metabolites on T cells which impairs their proliferation, and induction of iTreg capable of upregulating IDO expression in DCs through CTLA-4: B7 binding resulting in a suppressive phenotype (57). Suzuki et al. demonstrated significantly lower Trp concentrations in patient sera with known primary lung cancer in comparison to controls; IDO activity was significantly higher as measured by increased KYN/Trp ratios (58). The IDO1 pathway shifts the balance in the TME toward differentiation of naïve T cells into iTreg. This process requires activation of the aryl hydrocarbon receptor (AhR) (59). KYN is an endogenous ligand of AhR (60). Following activation of the AhR and translocation of the activated complex to naïve T cell nuclei, AhR complexes mediate changes in CpG methylation status of FoxP3 promoter resulting in increased FoxP3 expression (61). Naïve AhR–/– CD4+ T cells are not capable of undergoing conversion to FoxP3+ iTreg (62). The AhR pathway is highly active in human brain tumors and is associated with poor patient outcomes (60).

Harnessing IDO provides potential clinical opportunity to reverse anti-tumor suppression. Several first-in-man clinical trials were initiated to investigate safety, toxicity, and maximal biologic effects of single agent IDO1 inhibitors including indoximoid, INCB024360, and NLG919 in patients with various refractory solid tumor malignancies. Targeting IDO1 as a single agent therapeutic strategy failed to induce tumor regression (63, 64). However, there are several ongoing clinical trials using IDO1 inhibitors in conjunction with standard chemotherapeutic agents for breast, brain, pancreatic, and prostate tumors (63, 65, 66).

### Dynamic Transdifferentiation

Also in the TME, there is ongoing reprogramming of already differentiated populations of Treg and effector T cell populations toward other lineages (67). TGF-β, IL-2, and prostaglandin E_2_ (PGE_2_), found in the TME, are central in the reprogramming of Th17 cells toward a Treg phenotype (68). Il-17A^+^FoxP3+ T cells display phenotypic overlap with Treg and Th17 cells, and express equivalent amounts of CD25 and CCR4 (69). Populations in the TME are defined as IL-17A^+^FoxP3^+^ and ex-Th17 FoxP3+ cells converted from IL-17A^+^FoxP3^−^ cells (68). Human ovarian cancer ascites contains increased numbers of 17A^+^FoxP3+ T cells in comparison to matched patient blood samples indicating Th17 to Treg plasticity is driven by the TME (68). Additionally, IL-17A^+^FoxP3^+^ T cells are found in colorectal cancer human tissue samples, and have been demonstrated to induce colorectal cancer associated cell markers (70). Therefore, targeting this unique population of IL-17A^+^FoxP3^+^ T cells has the potential to restore effective anti-tumor immunity.

## Mechanisms of Treg-Mediated Suppression in the Tumor Microenvironment

Once in the TME, nTreg, and iTreg use mechanisms to carry out immunosuppression aiding in immune escape. Although there are distinctions between the ontogeny of nTreg and iTreg, deciphering the two populations *in vivo* is difficult, making it challenging to classify unique mechanisms of suppression between populations. Cancer literature describing Tregs in the TME infrequently distinguish between nTreg and iTreg. There likely is overlap between nTreg and iTreg suppressive mechanisms which will be discussed with the notion that there are shared functions between populations (71). In certain mouse models, both nTreg and iTreg are required for full induction of tolerance (72). There are two broad categories by which Treg deploy anti-tumor effects: contact dependent mechanisms which require cell binding, and contact independent mechanisms mediated through secretion of soluble products (73). Mechanisms of suppression are outlined in Table 2.

**Table 2:** Mechanisms of Treg suppression.

	**Proposed mechanism of action**	**References**
**CELL CONTACT DEPENDENT METHODS**		
CTLA-4: CD80/CD86	Blockade and down-regulation of co-stimulatory molecules on APCs thereby preventing T cell activation.	(74–77)
LAG-3:MHC II	LAG-3: MHC II interaction results in impaired maturation of DCs, and anergy and arrest of effector T cell populations.	(78, 79)
Nrp-1:MHC II	Nrp-1 on Treg facilitates prolonged interactions with DCs in an MHC II dependent fashion blocking access of effector T cells to APCs and downregulates DC costimulatory molecules.	(80, 81)
Granzyme/Perforin	Treg expression of perforin and granzyme targets NK and CD8+ T cells triggering target cell caspase-dependent apoptosis preventing immune response.	(82, 83)
**CELL CONTACT INDEPENDENT METHODS**		
IL-10 secretion	Suppression of IFNγ dependent activation of APCs, downregulation of MHC II and CD86 resulting in suboptimal T cell activation.	(84)
TGF β secretion	Downregulation of IL-2 required for lymphocyte survival and upregulation of cell cycle inhibitors resulting in cell cycle arrest of T cells.	(85)
IL-2 consumption	Treg expression of IL-2 Receptor (CD25) facilitates more efficient consumption of the cytokine leading to impaired T effector differentiation and survival signaling.	(86)
Generation of extracellular adenosine	Treg ectonucleotidases (CD39 & CD79) expressed on cell surface hydrolyzes ATP to adenosine mediating anti-inflammatory through T cell anergy, inhibition of pro-inflammatory cytokine production.	(87)
IL-35	Secretion by Treg results in inhibition of T cell proliferation through cell cycle arrest at the G1-S transition point.	(88, 89)

*nTreg and iTreg implement both contact dependent and contact independent mechanisms of immunosuppression in the TME*.

### Cell Contact Dependent

Contact dependent mechanisms include interactions between cognate receptors and ligands. CTLA-4 is a checkpoint inhibitor that is upregulated following T cell activation; it is constitutively expressed on Treg (74). CTLA-4 inhibits proliferation, cytokine production, and survival pathways of effector T cells through interactions with APCs (74, 75). CTLA-4 is a homolog of CD28; competitive binding of Treg CTLA-4 to CD80/86 on APC blocks CD28-mediated costimulatory signals, and downregulates other costimulatory molecules required for T cell activation (76). Ovcinnikovs et al. established that CTLA-4 captures costimulatory molecules CD80 and CD86 from APCs by transendocytosis, which then inhibits CD28 mediated costimulation of T cells. Treg specifically outperform effector T cells in their ability to transendocytose CD80 and CD86, and migratory DCs are targeted by Treg CTLA-4 *in vivo* (90). Onishi et al. demonstrate that nTreg preferentially aggregate on DCs *in vitro*, resulting in downregulation of DC activation markers, CD80 and CD86, thereby inhibiting maturation and ability to activate naïve T cells (91). A higher frequency of circulating CTLA-4+FoxP3+ Treg and MDSCs has been demonstrated in HCC blood samples (92). There is increased expression of CTLA-4 on intra-tumoral Treg in comparison to peripheral Treg (93). Treatment with anti-CTLA-4 antibody decreases tumor burden, Treg presence, and Treg to CD4+ and CD8+ ratios (93). Treatment also increases expression of proinflammatory cytokines including IFNγ, TNFα, and IL-13 in the TME (93). Mice vaccinated with irradiated B16 melanoma cells and treated with checkpoint signal blockade including anti-CTLA-4 and/or anti-PD-1resulted in marked resolution of solid tumor burden and increased CD4+/CD8+ to Treg ratio in the tumor (75). Therefore, Treg CTLA-4 is a dominant mechanism of immunosuppression that continues to promote inhibition of the anti-tumor response.

Lymphocyte activation gene−3 (LAG-3) is a cell surface molecule expressed on activated T cells, NK and B cells, and functions as an immune regulatory protein. It is a homolog for CD4+, which allows for binding to major histocompatibility complex II (MHC II) on DC populations (78). LAG-3 intracellular signaling and the specific mechanisms of immunosuppression still remain to be elucidated; however, immunosuppression is likely achieved through LAG-3: MHC II interaction resulting in impaired maturation of DCs, and anergy and arrest of tumor infiltrating T cell (78, 79). On activated Treg, LAG-3 expression becomes upregulated facilitating robust interaction with MHC II on DCs, further promoting suppression of the anti-tumor response in the TME (94). LAG-3+CD4+CD25+ Treg isolated from colorectal cancer patients secrete high levels of immunosuppressive cytokines including TGFβ and IL-10, thereby maintaining the immunosuppressive milieu (95). Camisaschi et al. describe a population of CD4+CD25+FoxP3+ T cells expressing LAG-3 with increased presence in peripheral circulation and solid tumor in advanced stage melanoma and colorectal cancers. LAG-3+ CD4+CD25+FoxP3+ Treg demonstrate enhanced suppressive capabilities vs. LAG-3- cells. When LAG-3+ Treg and CD4+CD25- T cells are separated via membrane, Treg suppressive capabilities are abrogated indicating that the mechanism required direct cell contact (96). Therefore, direct engagement of LAG-3 to MHC II molecules found on a number of cells in the TME provides an additional mechanism by which Treg achieve immunosuppression.

Perforin induces pore formation in membranes, allowing entry of granzymes A and B into the cytosol, triggering target cell caspase-dependent apoptosis (97, 98). Reports characterize Treg expression of perforin and granzyme as means of immunosuppression (99). Treg derived perforin and granzyme target NK and CD8+ T cells, rendering them incapable of eliminating pathologic tumor cells resulting in tumor expansion (100). Li et al. evaluated expression of granzyme B and perforin in Treg from the human breast cancer TME; Treg isolated from breast tissue consistently expressed significantly higher levels of granzyme B in comparison to Treg from peripheral blood samples of the same patient (82). Treg isolated from RMAS lymphomas or malignant ascites have significantly increased expression of granzyme B, but not granzyme A in comparison to splenic Treg or Treg from non-draining peripheral LNs of the same host animal. Granzyme B knockout (gzmb–/–) mice, which lack Treg expressing granzyme B, are more efficient in clearing RMAS implants in comparison to wild type controls. Therefore, elimination of Treg derived granzyme B allows for optimized NK and CD8+ T cell-mediated tumor control, with improved disease survival and outcomes (83). Conversely, inhibition of NK and CD8+ T cells in the TME by Treg derived perforin and granzyme results in tumor growth and progression. Administration of selective inhibitors of perforin, EGTA and concanamycin A, blocked Treg cytotoxic killing (101). Therefore, Treg-derived perforin and granzymes suppress cytotoxic lymphocytes in the TME, preventing tumor cell killing; elimination of Treg-derived perforin and granzyme results in activation of the antitumor immune response leading to tumor cell death and improved disease prognosis.

Nrp-1 is a transmembrane glycoprotein that serves as a co-receptor for class III/IV semaphorins, VEGFs, and TGFβ, and is implicated in processes including cell migration, angiogenesis, immunity, and cancer development (102). Nrp-1 has been identified on cell membranes of pDCs, vascular endothelial cells, and Tregs. In metastatic cervical cancer, Nrp-1 expression on Treg in the tumor draining lymph node (TdLNs) is far higher than without metastatic implants (103, 104). Nrp-1 expression is significantly upregulated on Treg isolated from peripheral blood of chronic lymphocytic leukemia patients in comparison to healthy controls (80). Increased expression of Nrp-1 on Treg in cancer enhances immune suppression through interactions with DCs (81). To characterize this interaction, Sarris et al. use time lapsed video microscopy to quantify length of binding between immature DCs (iDCs) and CD4+CD25+FoxP3+ T cells which demonstrated frequent and prolonged interactions in an Nrp-1: MHC II dependent fashion. Nrp-1 also interacts in a homotypic fashion, allowing for prolonged Treg and DC binding (105). Prolonged interaction between the Treg:DC populations blocks access of effector T cells to APCs and downregulates DC costimulatory molecules (106). Without costimulation, T cells become anergic, resulting in failure of the anti-tumor immune response. In a melanoma model, conditional knock out of Nrp-1 resulted in impaired tumor growth with a decrease in intratumoral Tregs (107). Together, Nrp-1 is required for Treg stability in the TME and facilitates interaction with other infiltrating immune cells, preventing activation of a robust anti-tumor immune response (108).

### Contact Independent Mechanisms

Treg also employ contact independent mechanisms of suppression mediated through secretion of inhibitory cytokines and local competition for growth factors (109).

#### Cytokines

Anti-inflammatory cytokines are integral in maintenance of homeostasis and prevention of inflammatory immune responses. IL-10 is an anti-inflammatory cytokine responsible for maintenance of self-tolerance, but also inhibition of the anti-tumor immune response. IL-10 mediates immunosuppression through several mechanisms, including suppression of IFNγ dependent activation of APCs with decreased expression of MHC II and CD86, preventing optimal T cell activation (84). IL-10 sustains expression of FoxP3, TGFβR, and TGFβ by recently activated Treg, stabilizing their suppressive phenotype (110). IL-10 mRNA has been isolated from fresh human tumors including ovarian, breast, renal cell, lung, and squamous cell carcinoma (40). Treg have been identified as a major source of IL-10 in the TME. Stewart et al. identified increased IL-10 expression in CD4+CD25+FoxP3+ intratumoral Treg which demonstrated a highly activated suppressor phenotype (111). In patients with HNSCC, tumor infiltrating Treg consistently expressed GITR, FasL, TGFβ, and IL-10. Immunohistochemistry demonstrated IL-10 expression by CD4+CD25+ T cells, but not by tumor associated macrophages or DCs (112). Peripherally circulating and intratumoral Treg in gastric cancer patients express greater quantities of IL-10 in comparison to CD4+ CD25- T cell populations (113). IL-10 in the TME and peripheral blood portends poorer disease prognosis for ovarian cancer, more advanced stage disease in colorectal cancer, and increased tumor diameter in NSCLC (114–116). Altogether, IL-10 serves as an immunosuppressive agent, with effects on many cell types in the TME, that could serve as a therapeutic target in cancer therapy.

TGFβ achieves its immunosuppressive functions via several effects, including downregulation of IL-2 which is a requirement for lymphocyte survival, upregulation of cell cycle inhibitors resulting in cell cycle arrest and impaired T cell proliferation, and control of expression of effector molecules (85). TGFβ in the TME is implicated in poor disease prognosis, later stage disease, and LN metastasis in cancers including gastric, breast, and colon carcinoma (117–119). In mice inoculated with 4T1 mammary carcinoma, treatment with cyclophosphamide and anti- TGFβ mAb decreased tumor growth, resulted in massive infiltration of IFNγ producing lymphocytes, and upregulated MHC II and CD80 on APCs. Following combination therapy, mice were also resistant to tumor re-challenge indicating development of a durable anti-tumor response (120).

IL-35 is an inhibitory cytokine that plays a role in the TME. It is a heterodimeric member of the IL-12 family composed of the p35 subunit of IL-12 and the Ebstein Barr virus induced gene 3 subunit. IL-35 is secreted by mouse and human Treg, and is required for regulatory activity *in vitro* and *in vivo* (121, 122). IL-35+ cell types have been isolated from tumor bearing mice and human cancer samples including acute myeloid leukemia, prostate, and colorectal cancer (122–124). IL-35 intracellular signaling is highly variable depending on the particular immune cell type but is in part mediated through the JAK-STAT pathway, resulting in inhibition of T cell proliferation through cell cycle arrest at the G1-S transition point (88, 89). In patients with pancreatic ductal adenocarcinoma, IL-35 secreting Tregs are more prevalent in the peripheral blood, TME, and TdLNs in comparison to healthy controls. Increased IL-35 plasma concentrations positively correlate with increased tumor size and later stages (125). In the B16 melanoma model, IL-35 blockade resulted in inhibition of tumor growth, increased infiltration of CD4+ and CD8+ effector T cells in tumor tissue, TdLNs, and non-TdLNs. Infiltrating lymphocytes from IL-35 neutralized mice had an activated effector phenotype (126). Therefore, IL-35 could serve as a potential therapeutic target in the TME to prevent inhibition of tumor specific infiltrating T lymphocytes.

#### Local Competition for Growth Factors

IL-2 is a pleiotropic cytokine with an array of functions and activities specific to the TME (86). IL-2 has a number of effects on infiltrating CD4+ and CD8+ T cells which include expansion and proliferation of antigen specific clones, enhanced secretion of pro-inflammatory cytokines, and augmentation of cytolytic activity (127). Interestingly, mice with targeted deletions of IL-2 or IL-2 receptor subunits develop severe autoimmune phenotypes indicating an additional important role of IL-2 in tolerance (127). Treg constitutively express the high affinity IL-2 receptor (IL-2R), otherwise known as CD25, which drives the survival and population expansion of Foxp3+ Treg (128). Although IL-2 is required for Treg survival, it is not a requirement for its specific suppressive functions (129). Pandiyan et al. demonstrate that Treg use high affinity CD25 to out-compete surrounding responder T cells for IL-2 resulting in responder T cell apoptosis, a phenomenon known as IL-2 sinking (130). Therefore, anti-tumor effector IL-2 deprivation by Treg in the TME promotes tumor tolerance through limitation of effector T cell responses.

#### Generation of Extracellular Adenosine

Adenosine triphosphate (ATP) is readily present in the TME as a result of ongoing cellular stress, plasma membrane damage, and hypoxia. ATP enters the TME through exocytosis and active transmembrane transport where it is further metabolized into the highly immunosuppressive metabolite, adenosine (87). In the TME, multiple cellular constituents have the machinery to generate adenosine, most notably Treg (131) Conversion of ATP to adenosine is carried out by a membrane bound ectonucleotidases CD39 and CD73. Generated adenosine then targets infiltrating effector CD4+ and CD8+ T cells through the A2a receptor resulting in increased intracellular cAMP and inhibition of the pro-inflammatory NFκB pathways (132) Manapathil et al. demonstrate that Treg isolated from HNSCC patients highly express CD39 and CD73 in comparison to healthy controls. HNSCC derived Treg hydrolyze ATP at higher rates producing greater levels of adenosine vs. healthy counterparts. Increased ATP hydrolysis correlated with increased adenosine mediated effector T cell suppression, and more advanced stage disease (133). Targeted genetic deletion of CD73 in mice resulted in suppression of tumor growth and increased frequency of tumor antigen specific CD8+ T cells both in peripheral circulation and tumor tissue (134). Therefore, adenosine produced by Treg in the TME is highly immunosuppressive, and readily dampens effective effector immune response resulting in ongoing tumor evasion.

## Migration of nTreg and iTreg From TME

Following induction or activation, nTreg and iTreg migrate out of the TME to further carry out immunosuppressive functions. TdLNs serve as an extension of the TME, further facilitating tumor growth and metastasis. Our lab has investigated Treg homing patterns in a transplantation model, and have demonstrated the role of the lymphotoxin-beta receptor (LTβR)/lymphotoxin (LTα1β2) pathway (135). Without suitable Treg trafficking from allografts to graft draining LNs (dLNs), there is inadequate development of a tolerogenic immune response that is not overcome with Treg migration from blood to graft dLNs (135). This suggests the presence of Tregs in the tumor dLN as a requirement for propagation of the anti-tumor immune response.

The exact mechanisms by which iTreg or nTreg are enticed to leave the tumor site and engage in migration to the tumor dLN requires further characterization. Deng et al. demonstrated increased numbers of CD4+CD25+ FoxP3+ cells within TdLNs of colorectal cancer patients compared to CD4+CD25+FoxP3- cells. This trend was more apparent in samples obtained from advanced stage disease; therefore, Treg presence in TdLNs can be used as a correlate for overall disease progression (136). Lee et al. demonstrated an increased population of FoxP3+ T cells within the sentinel LN of gastric cancer patients, correlating with the increased occurrence of down-stream LN metastasis. Increased presence of Tregs in the sentinel LN of breast cancer patients is strongly correlated with clinically undetectable micro-metastatic disease (137). Therefore, FoxP3+ infiltration into dLN can serve as a prognostic indicator of LN metastasis (138).

## Non-Immunologic Treg and Tumor Interactions

Various interactions have been described above detailing the interplay between the TME and recruitment or induction of Tregs within tumors, and resulting changes in immune responses. However, fewer studies detail direct physiologic benefits tumors derive from Treg related to nonimmunologic effects including tumor viability, growth, metabolism, metastasis, and survival. Tregs appear to have pro-tumor influences, and provide proof of concept for direct Treg-tumor interactions that are beneficial to tumor physiology.

Intratumoral hypoxia drives angiogenesis and lymphangiogenesis which is induced by oxygen overconsumption of rapidly dividing tumor cells which readily outgrow blood supply and lymphatic drainage (139). Lymphangiogenesis and angiogenesis in the TME are orchestrated by tumor cells and tumor infiltrating lymphocytes through release of cytokines and growth factors in response to hypoxia and nutritional depletion (140). Hypoxia leads to increased transcription of vascular endothelial growth factors (VEGFs) including VEGF-C and D, which target lymphatic endothelial cells (LECs) expressing VEGF receptors (VEGF-Rs) including VEGFR3. Lymphangiogenesis is induced via LEC proliferation, sprouting, and migration, thereby facilitating cancer progression and metastasis (139). CD4+CD25+ Tregs facilitate neovasculature development; Treg secrete larger quantities of VEGFA in both basal and hypoxic conditions compared to CD4+CD25- T cells (13, 14). Hypoxic Treg conditioned medium induces formation of tube-like capillary structures, with increased lengthening of capillary endothelial networks of human umbilical vein endothelial cells compared to CD4+CD25- T cells. This process is VEGF dependent, and blockade of VEGFR1/2 results in a depressed pro-angiogenic response (14). Therefore, Treg home to the TME via a number of previously described mechanisms, secrete VEGFs, and facilitate tumor survival and progression through induction of angiogenesis and lymphangiogenesis.

Foxp3+CD4+ Treg are present in non-lymphoid structures including skeletal muscle, visceral adipose tissue, and the colonic lamina propria (141). These Treg function in an equivalent manner to Treg derived from secondary lymphoid tissue in *in vitro* suppression assays (142). However, tissue specific Tregs transcriptomes have unique characteristics when compared to lymphoid derived Treg (141). Thus, tumor derived Treg may represent a distinct subset of tissue Treg with properties, not only capable of suppressing the immune response, but also enhancing cancer growth and metastasis. For example, skeletal muscle Treg home to injured muscle in acute and chronic injury, and repair myocytes through a series of events including activation, proliferation, differentiation, migration, and formation of myofibers (143). This process is fueled by the interaction between IL-33, and its transmembrane receptor, ST2 (144). IL-33 is highly expressed by acutely injured skeletal muscle due to cellular stress and injury (145). IL-33 expression has also been documented in the TME with increased expression in HNSCC, gastric cancer, NSCLC, HCC, and breast cancer (146). IL-33 functions as a tissue alarmin indicating tissue necrosis and destruction (147). IL-33 is capable of remodeling the TME and stromal constituents, facilitating tumor growth and metastasis. IL-33 favors tumor tolerance through inducing M2 macrophages, activating MDSCs, and Tregs (148). ST2 has been identified on the surface of tissue repair Treg at higher levels than lymphoid counterparts (149). ST2+ Treg promote tissue repair after receptor activation by secreting amphiregulin (AREG). Increased AREG results in *in vivo* muscle regeneration (150). Although the IL-33:ST2 interaction in the TME and its effects on Treg mediated tumor cell repair have not been reported, we speculate that increased IL-33 expression by tumor cells and surrounding stromal cells potentiates Treg homing capabilities to the TME in a ST2 dependent manner, where infiltrating Treg are then capable of initiating tissue repair and tumor cell survival.

In injured skeletal muscle, Treg facilitate repair through pathways related to AREG. AREG is a member of the epidermal growth factor (EGF) family and signals through epidermal growth factor receptor (EGFR), notably found on muscle cells and tumor cells including colon, breast, prostate, pancreatic, bladder, ovarian, and melanoma (151). EGFR activation induces events that determine cell fate, proliferation, differentiation, and development of tumors. Treatment with EGFR inhibitors has promising success related to blockade of survival and growth signals needed by tumors for progression (152). The AREG-EGFR axis also promotes increased cell motility and invasion. The MDA-231 breast cancer cell line has high basal expression of AREG and TGFα, both ligands for EGFR. Knockdown of EGFR, AREG or TGFα expression resulted in decreased tumor cell motility, slower growing tumor cells, and increased survival. Overexpression of AREG, TGFα or both ligands increased tumor burden, increased tumor vascularity, increased number of infiltrating macrophages, and portended poorer survival (15). Although it has not been reported for the TME, Treg have the capacity to secrete large quantities of AREG upon optimal activation (152). Increasing numbers of Treg secreting AREG in skeletal muscle of mice with muscular dystrophy results in enhanced muscle regeneration (143). Further investigations will be required to assess AREG produced by tumor infiltrating Treg. We speculate that AREG-EGFR interactions in the TME facilitate tumor growth and invasion. AREG-EGFR signaling may be important in tumor proliferation and repair in the TME.

Epithelial mesenchymal transition (EMT) is critical for metastatic transition, allowing for tumor cells to lose polarity through loss of cell-cell contact, become more invasive, and become resistant to apoptosis (153). EMT promoting transcription factors Slug, Snail, and Zeb 1 and 2 disrupt transcription of E-cadherin and occludins, which maintain cell polarity and adherence (154). Treg function as a central regulator of this process as a source of TGFβ in the TME, that make tumor cells more prone to metastasis (16). Mammary epithelial cells (NMuMG) treated *in vitro* with TGFβ1 demonstrated increased expression of genes related to the Erk signaling pathway including *H-ras, N-ras, MEK2*, and *Erk1*. Cellular morphology altered to that of aberrantly elongated cells with loss of epithelial markers including zonulin-1 and E-cadherin from cell junctions (17). Inhibition of the Erk signaling pathway with a MEK1/2 inhibitor resulted in blockade of these morphological changes (17). Treg directly influence EMT in a pulmonary fibrosis model simulating thoracic radiation in lung cancer. Following exposure to 20 Gy thoracic radiation, mouse lung tissues develop characteristics of EMT, including alveolar septal thickening, loss of epithelial cell marker pro-surfactant protein C, and increase in mesenchymal marker N-cadherin (155). Thoracic radiation in conjunction with Treg depleting anti-CD25 antibody inhibited markers of mesenchymal transition, including less collagen deposition and minimal N-cadherin expression (155). Although the exact mechanisms by which Treg interact with surrounding epithelial cells or tumor tissues has not yet been defined, these observations demonstrate a relationship between EMT and Treg that is relevant to the TME.

## Selective Depletion of Tumor-infiltrating Treg

Given the strong correlation between tumor-infiltrating Treg in solid cancer and patient survival (23, 156–159), there is interest to deplete tumor-infiltrating Treg. Given the role for Treg in protection against autoimmune diseases (2, 160), it is imperative to selectively deplete only tumor-infiltrating Treg. Recent studies have demonstrated that this is achievable by targeting CTLA-4 molecules. While CTLA-4 is constitutively expressed on Treg both in and outside cancer tissues, cell surface CTLA-4 is minimally detectable among circulating Treg and those in lymphoid organs (161). In contrast, tumor-infiltrating Treg express high levels of cell surface and intracellular CTLA-4 (93, 162). This allows selective enrichment of systemically administered anti-CTLA-4 antibodies in tumor tissues (163).

In preclinical models, anti-CTLA-4 antibodies cause tumor rejection by engaging Fc receptors which are critical for antibody-dependent cell-mediated cytotoxicity (ADCC) or antibody-dependent cell-mediated phagocytosis (ADCP). We used human *CTLA-4* gene knock in mice (164) to show that tumor rejection mediated by Ipilimumab, an FDA-approved anti-CTLA-4 antibody, can be abrogated by an antibody that blocks interaction between IgG Fc and FcgRII and III (162). Consistently, two laboratories have demonstrated that the therapeutic effect of anti-mouse CTLA-4 antibodies is abrogated by targeted mutation of genes encoding either FcγRIV or activating FcγR (165, 166). In mice with humanized Fc receptors, a strong correlation was found between the ADCC activities of human IgG Fc isotypes and tumor-rejection induced by chimeric anti-CTLA-4 antibodies (167), which is consistent with earlier studies by Selby using an anti-CTLA-4 antibody in which the Fc portion incorporated various mouse IgG isotypes (93). Clinical data revealed that FcγRIIIA polymorphisms, which affect ADCC activity, strongly associate with therapeutic effect of Ipilimumab in melanoma patients (167).

Two groups have shown that Ipilimumab selectively reduces intratumoral Treg, but not those in the circulation (168, 169). While a third group did not find reduction of absolute Treg numbers in cancer tissues, the relative ratio of FOXP3+ cells over CD4+ T cells among patients receiving Ipilimumab was reduced when compared with either pre-treatment biopsy samples or compared with those that received another anti-CTLA-4 antibody, Tremelimumab, which is IgG2 isotype with less ADCC potential (167, 170) and has not been shown to be effective in phase III clinical trials (171).

In addition to targeted tumor specific Treg depletion via CTLA-4 blockade, other pharmacologic targets have been demonstrated to yield improved degrees of tumor rejection and restoration of the anti-tumor immune response. Monotherapy with CTLA-4 or PD-1 alone potentially leave other critical immune checkpoints unopposed leading to undesired upregulation of compensatory pathways on Treg (172). Curran et al. demonstrated that combination therapy including both CTLA-4 and PD-1 pharmacologic blockade resulted in significant reduction of pre-established melanoma tumor burden with associated restoration of a highly advantageous intratumoral T effector cell to Treg ratio (75). Similar findings are mirrored in a mouse glioma model; intra-cranial injection of CTLA-4 and PD-L1 mAb and IDO blockade resulted in enhanced survival with an associated decrease in infiltrating antigen experienced Treg, and an increase in cytolytic T cell presence (173). Clinical trials highlighting combination therapy of CTLA-4 and PD-1 or PD-L1 blockade demonstrated superior clinical efficacy to monotherapy in human melanoma (174, 175). Therefore, dual pharmacologic blockade successfully blocks negative costimulatory pathways allowing for successful activation of tumor specific effector T cell populations (75).

## Conclusions and Perspectives

nTreg and iTreg play roles in survival and growth of many cancer subtypes. Blunting of anti-tumor innate and adaptive immunity results in disease progression and poorer disease outcomes. nTreg home to the TME via chemokine gradients, and use receptor and cognate ligand interactions to aid in their arrival at the site of ongoing inflammation. iTreg are induced in the TME or in the dLN, and provide support for ongoing suppression in response to the cytokine milieu. nTreg and iTreg are derived via different pathways, but likely have overlapping specificities and mechanisms of suppression, resulting in deactivation and inhibition of host infiltrating immune cells. Further investigation on the relative contributions of both nTreg and iTreg on anti-tumor immunosuppression in the TME is required. Cell surface markers identifying the populations are not fully agreed upon making it fairly difficult to reliably differentiate between populations in the TME (9). Aside from the hallmark cell contact dependent and contact independent pathways of immunosuppression, there are non-immunological benefits that tumor cells derive directly from the presence of Treg in the TME. Treg play a part in lymphangiogenesis and angiogenesis, tumor cell motility, and EMT resulting in tumor survival, growth, and metastasis.

Therapeutic inhibition of Treg in the TME requires balance between optimization of the antitumor immune response, and the deleterious loss of self-tolerance. An understanding of the complex interactions taking place in the TME and associated TdLNs between Treg and tumor infiltrating immune cells provides significant opportunity for research. Development of novel approaches to prevent homing or induction of Treg in the TME will prevent accumulation of suppressive cytokines and allow for robust activation of infiltrating lymphocytes. Mechanisms to prevent Treg egress from the TME should be investigated to prevent Treg homing to TdLNs where the immunosuppressive milieu is propagated through inhibition of APCs. A comprehensive understanding of the complex non-immunologic benefits derived by tumors from Treg interactions require investigation to understand the multifaceted ability of tumor cells to adapt, survive, proliferate, and metastasize. Advances suggest that a new generation of anti-CTLA-4 antibodies can selectively deplete Treg in TME without affecting the number and function of Treg outside of cancer tissues (176). These data may inspire a new wave of clinical investigation that will provide important insights on clinical benefits of eliminating immune suppression of cancer by Treg.

## Author Contributions

CP and JB conceived of the presented manuscript idea. CP primarily authored the manuscript including literature review and figure and table development. JB directed ongoing paper review and editing. XC, RA, PZ, and YL discussed and contributed to the final manuscript editing.

### Conflict of Interest

The authors declare that the research was conducted in the absence of any commercial or financial relationships that could be construed as a potential conflict of interest.

## Abbreviations

ADCC: Antibody-Dependent Cell-Mediated Cytotoxicity
ADCP: Antibody-Dependent Cell-Mediated Phagocytosis
AhR: Aryl Hydrocarbon Receptor
APC: Antigen Presenting Cells
AREG: Amphiregulin
ATP: Adenosine Triphosphate
BCNU: bis-chloroethylnitrosourea
CLL: Chronic Lymphocytic Leukemia
CTLA-4: Cytotoxic T-Lymphocyte Associated Protein 4
DCs: Dendritic Cells
dLNs: Draining Lymph Nodes
EGFs: Epidermal Growth Factors
EGFR: Epidermal Growth Factor Receptor
EMT: Epithelial Mesenchymal Transition
FasL: Fas Ligand
FoxP3: Forkhead Box P3 Protein
GITR: Glucocorticoid Induced TNFR-Related Gene
Gzmb–/–: Granzyme B Knockout
HCC: Hepatocellular Carcinoma
HIF-1α: Hypoxia Inducible Factor 1α
HNSCC: Head and Neck Squamous Cell Carcinoma
iDCs: Immature Dendritic Cells
IDO: Indoleamine 2,3-Dioxygenase
IL-2 R: IL-2 Receptor
IL-10 R: IL-10 Receptor
iTreg: Induced T Regulatory Cells
KYN: Kynurenine
LAG-3: Lymphocyte Activation Gene−3
LECs: Lymphatic Endothelial Cells
LNs: Lymph Nodes
LTβR: Lymphotoxin Beta Receptor
LTα1β2: Lymphotoxin Alpha 1 beta 2
mAb: Monoclonal Antibody
MDSCs: Myeloid Derived Suppressor Cells
MHC I & II: Major Histocompatability Complex I & II
MO-MDSCs: Monocytic Myeloid Derived Suppressor Cells
MVs: Microvesicles
NSCLC: Non-Small Cell Lung Cancer
nTreg: Natural T Regulatory Cells
Nrp-1: Neuropilin-1
PD-1: Programmed Death Receptor-1
pDCs: Plasmacytoid Dendritic Cells
PD-L1/PD-L2: Programmed Death Ligand−1 & 2
PGE2: Prostaglandin E2
S1P: Sphingosine-1-Phosphate
S1PR1: Sphingosine-1-Phosphate Receptor-1
TdLNs: Tumor Draining LNs
TGFβ: Transforming Growth Factor β
TGFβ- R: Transforming Growth Factor β Receptor
TME: Tumor Microenvironment
Treg: T Regulatory Cells
Trp: Tryptophan
VD3, 1α: 25-Dihydroxyvitamin D3
VEGFs: Vascular Endothelial Growth Factors
VEGFRs: Vascular Endothelial Growth Factor Receptors.
